# The complete chloroplast genome data of *Bergera glabra* (Guillaumin) F.J.Mou, a potential aromatic plant endemic to Vietnam

**DOI:** 10.1016/j.dib.2026.113313

**Published:** 2026-10-06

**Authors:** Nhat Nam Nguyen

**Affiliations:** School of Agriculture and Aquaculture, Tra Vinh University, Vinh Long, 87000, Vietnam

**Keywords:** Curry tree, Endemic taxa, *Murraya glabra*, Taxonomic revision, Rutaceae

## Abstract

The genus *Bergera* J.Koenig is renowned for the curry tree (*Bergera koenigii* L.) and related aromatic species. However, most available genomic data for this genus have focused on *B. koenigii* because of its widespread use as a spice. In this study, we sequence and report the complete chloroplast genome of *Bergera glabra* (Guillaumin) F.J.Mou, a species endemic to Vietnam. The reported genome has a quadripartite structure, comprising a large single-copy region (87,759 bp and 37.1% GC), a small single-copy region (18,075 bp and 33.7% GC), and two inverted repeat regions (27,112 bp and 43% GC each). Additionally, the genome contains 79 protein-coding genes, 30 transfer RNA genes, and four ribosomal RNA genes. Phylogenetic analysis indicated the monophyly of the genus *Bergera* and a close relationship between *B. glabra* and *Bergera euchrestifolia* (Hayata) F.J.Mou*.* This study expands the genomic data of the genus *Bergera* and provides useful information for further genomic studies examining population genetics and molecular markers for *B. glabra* and other related *Bergera* taxa.

Specifications Table

**Table:** 

Subject	Biology
Specific subject area	Genomics: Chloroplast genome
Type of data	Figure Raw, Analyzed
Data collection	Total genomic DNA of *Bergera glabra* was extracted from fresh, healthy leaves using a commercial kit. The Illumina NovaSeq system was employed for sequencing. Subsequently, fastp v1.3.6 and NOVOPlasty v4.3.5 were used to filter the raw reads and assemble the complete chloroplast genome, respectively. Next, GeSeq and OGDRAW were used to annotate and visualize the newly assembled chloroplast genome, respectively. The complete chloroplast genomes of 40 Rutaceae species and three outgroups were used to reconstruct phylogenetic relationships. The chloroplast genomes were aligned using MUSCLE v5, and the resulting data matrix was used to conduct phylogenetic analysis using IQ-TREE v2.4.0 and MrBayes v3.2.7a. FigTree v1.4.5 was used to modify and illustrate the phylogenetic tree.
Data source location	Location: Medicinal Plant Garden of Tra Vinh University, Vinh Long, Vietnam (9°55′24.7″N 106°20′47.4″E, altitude: 1–2 m). Voucher information: The specimen of *B. glabra* was deposited in the School of Agriculture and Aquaculture, Tra Vinh University, under voucher number NHTI-NTTU–20,251,206-003 (contact person: Nguyen Nhat Nam, email: nnnam@tvu.edu.vn).
Data accessibility	Repository name: National Center for Biotechnology Information (NCBI)Direct URLs to the complete chloroplast genome and raw data. 1. https://www.ncbi.nlm.nih.gov/nuccore/PZ698166 2. https://www.ncbi.nlm.nih.gov/bioproject/?term=PRJNA1516684 3. https://www.ncbi.nlm.nih.gov/sra/?term=SRR40302717 Repository name: FigshareDirect URL to data for phylogenetic analysishttps://doi.org/10.6084/m9.figshare.33295419
Related research article	NONE

## 1 Value of the Data

• The raw dataset contains nuclear, mitochondrial, and chloroplast genomic sequences, providing a valuable resource for future genomic studies of *B. glabra* that examine genome sequences, structure, and evolution.
• The features of the *B. glabra* chloroplast genome, including genome size, genome structure, and gene content, provide fundamental genomic data for further studies characterizing the chloroplast genome, tracing evolutionary history, and developing molecular markers for *B. glabra* and other *Bergera* species.
• Phylogenetic relationship analysis based on complete chloroplast genomes confirmed the monophyly of the genus *Bergera* and the taxonomic position of *B. glabra*, which was previously separated from the genus *Murraya*.

## 2 Background

*Bergera glabra* (Guillaumin) F.J.Mou, a member of the family Rutaceae, is endemic to Vietnam and has been reclassified as a member of the genus *Bergera* rather than the genus *Murraya* based on molecular data, including ITS, *trnL-F, atpB-rbcL, matK, psbH-petB*, and *psbA-trnH* sequences, and morphological data, including whole plant, leaf, inflorescence, and fruit characteristics [1,2]. Previously, the essential oil extracted from the leaves of *B. glabra* was characterized by its mosquito larvicidal and antimicrobial activities [3]. However, the chloroplast genome of *B. glabra* remains unexplored. In contrast, various aspects of other *Bergera* species have been studied, such as the curry tree (*Bergera koenigii* L.), of which phytochemical content exhibited antioxidant, antimicrobial, antidiabetic, antiobesity, and anti-inflammatory activities [[4], [5], [6]]. Additionally, chloroplast genome and transcriptomic data of *B. koenigii* have been analyzed [7,8]. Recently, complete chloroplast genomes of four *Bergera* species distributed in China have been reported and compared with those of other Rutaceae species to elucidate phylogeny and estimate divergence time [9]. In the current study, we report the complete chloroplast genome of *B. glabra* based on next-generation sequencing method (Fig. 1). This new dataset provides the first characterized organellar genome of *B. glabra* and adds valuable data for future studies on the evolutionary history and population genetics of the genus *Bergera*.

**Fig. 1:**
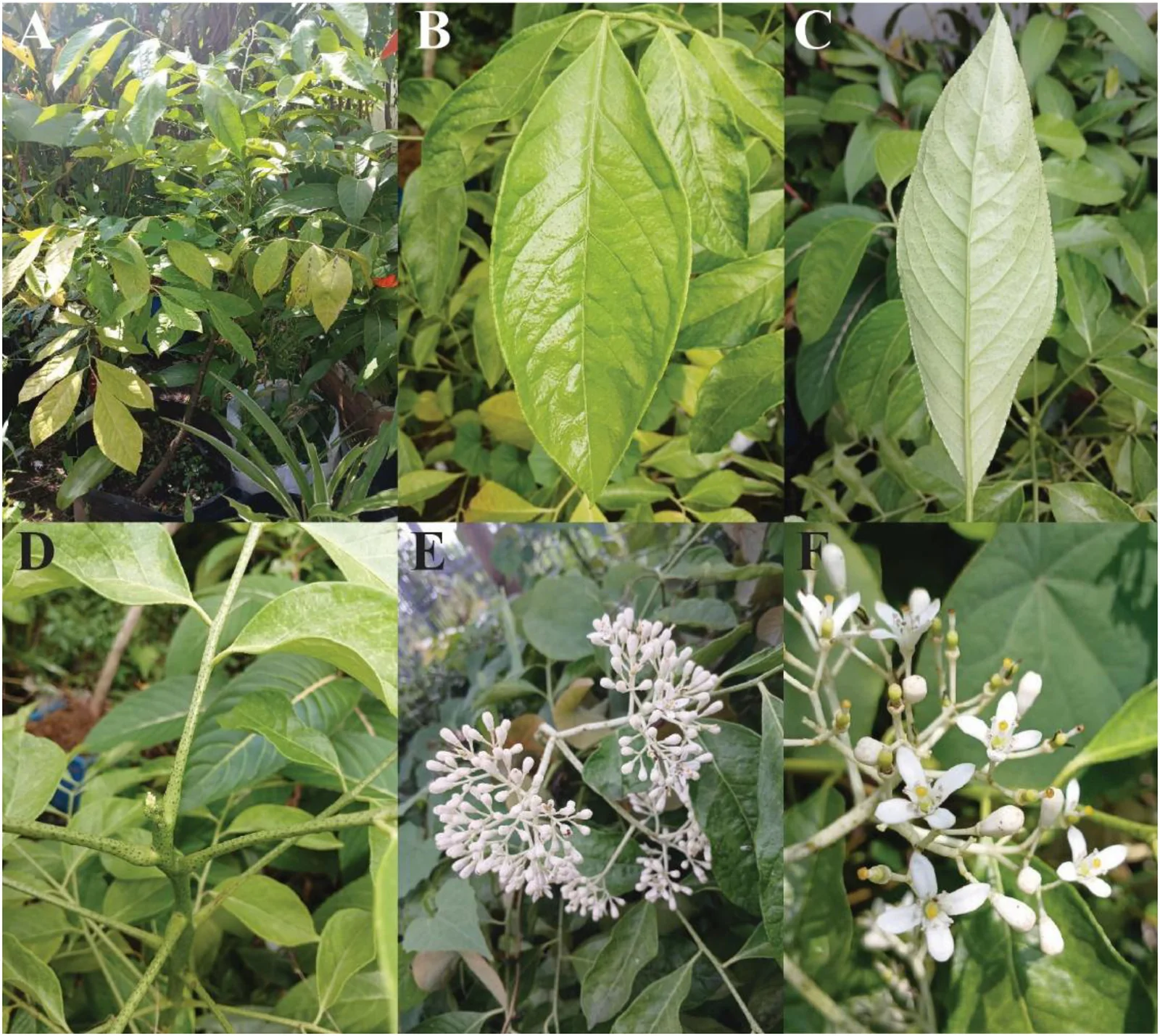
Photos of *Bergera glabra*. A. Whole plant. B. Upper leaf surface. C. Lower leaf surface. D. Young shoot. E. Inflorescence. F. Blooming flower. A shrub or small tree with oil glands in branches, rachis, petiole, and petals. Compound obovate or oblong-lanceolate leaves. Terminal cymes with many small, four-angled white flowers.

## 3 Data Description

To complete the chloroplast genome of *B. glabra*, there were 10,679,991 of 439,575,678 raw reads utilized, achieving an average coverage of 13,465× (Fig. 2). This genome was 160,058 bp in length with a GC content of 38.7% and comprised a large single-copy region (87,759 bp and 37.1% GC), a small single-copy region (18,075 bp and 33.7% GC), and two inverted repeat regions (27,112 bp and 43% GC each) (Fig. 3). The annotation results revealed 79 protein-coding genes, 30 transfer RNA genes, and four ribosomal RNA genes in the chloroplast genome of *B. glabra* (Fig. 3). Among the 79 protein-coding genes, 15 genes contained one intron (such as *atpF, ndhA, ndhB, petB, petD, ps16, rpl16, rpl2, rpoC1, trnA_UGC, trnG_UCC, trnI_GAU, trnK_UUU, trnL_UAA*, and *trnV_UAC*), while two genes had two introns (*pafI* and *clpP1*). The presence of two inverted repeat regions resulted in the duplication of 20 genes (including *ndhB, rpl2, rrn4.5, rrn5, rrn16, rrn23, rpl22, rpl23, rps7, rps12, rps19, trnA_UGC, trnI_CAU, trnI_GAU, trnL_CAA, trnN_GUU, trnR_ACG, trnV_GAC, ycf1, and ycf2*) but *ycf1* was incompletely duplicated.

**Fig. 2:**
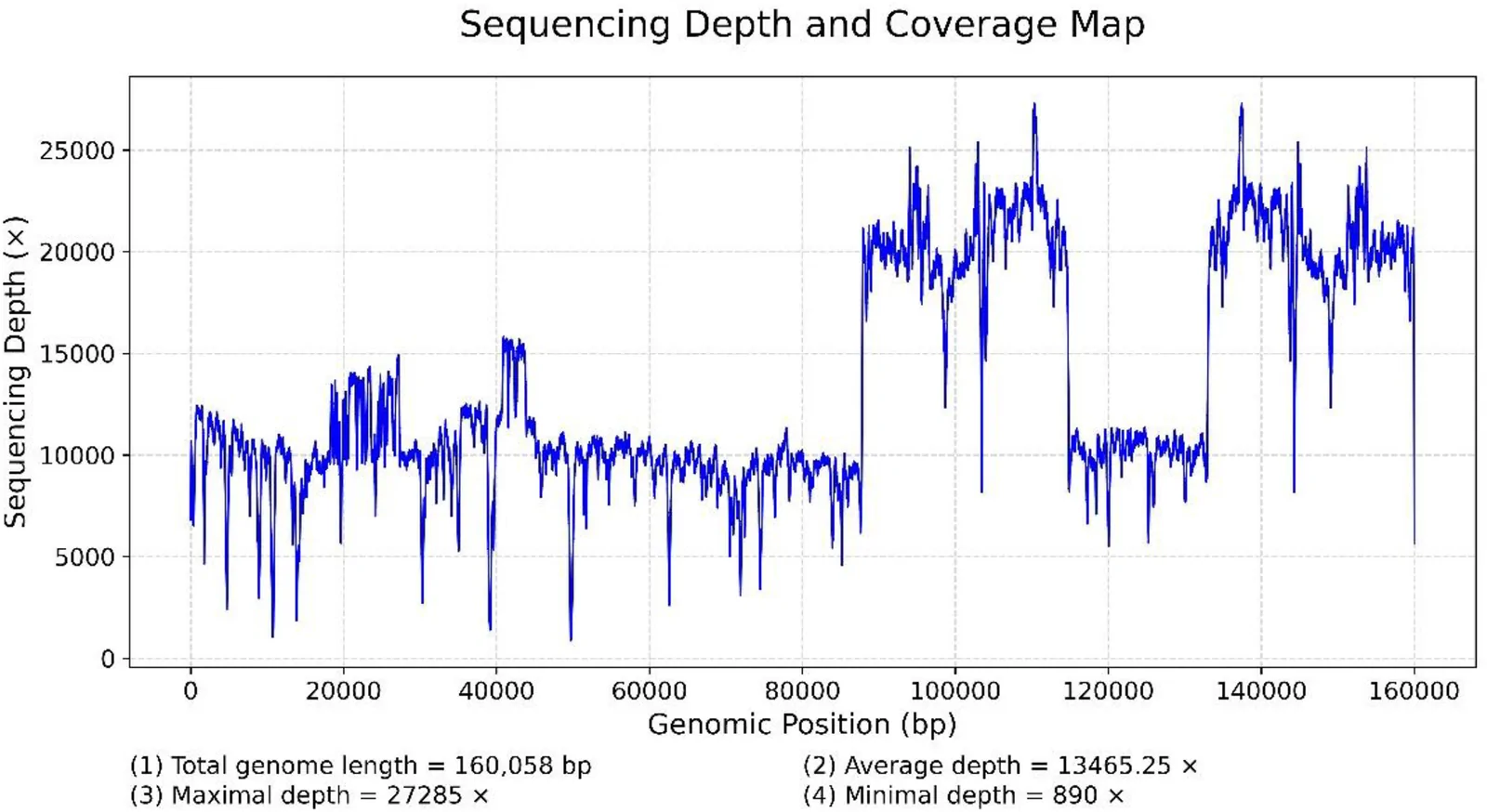
Coverage depth of the *Bergera glabra* chloroplast genome.

**Fig. 3:**
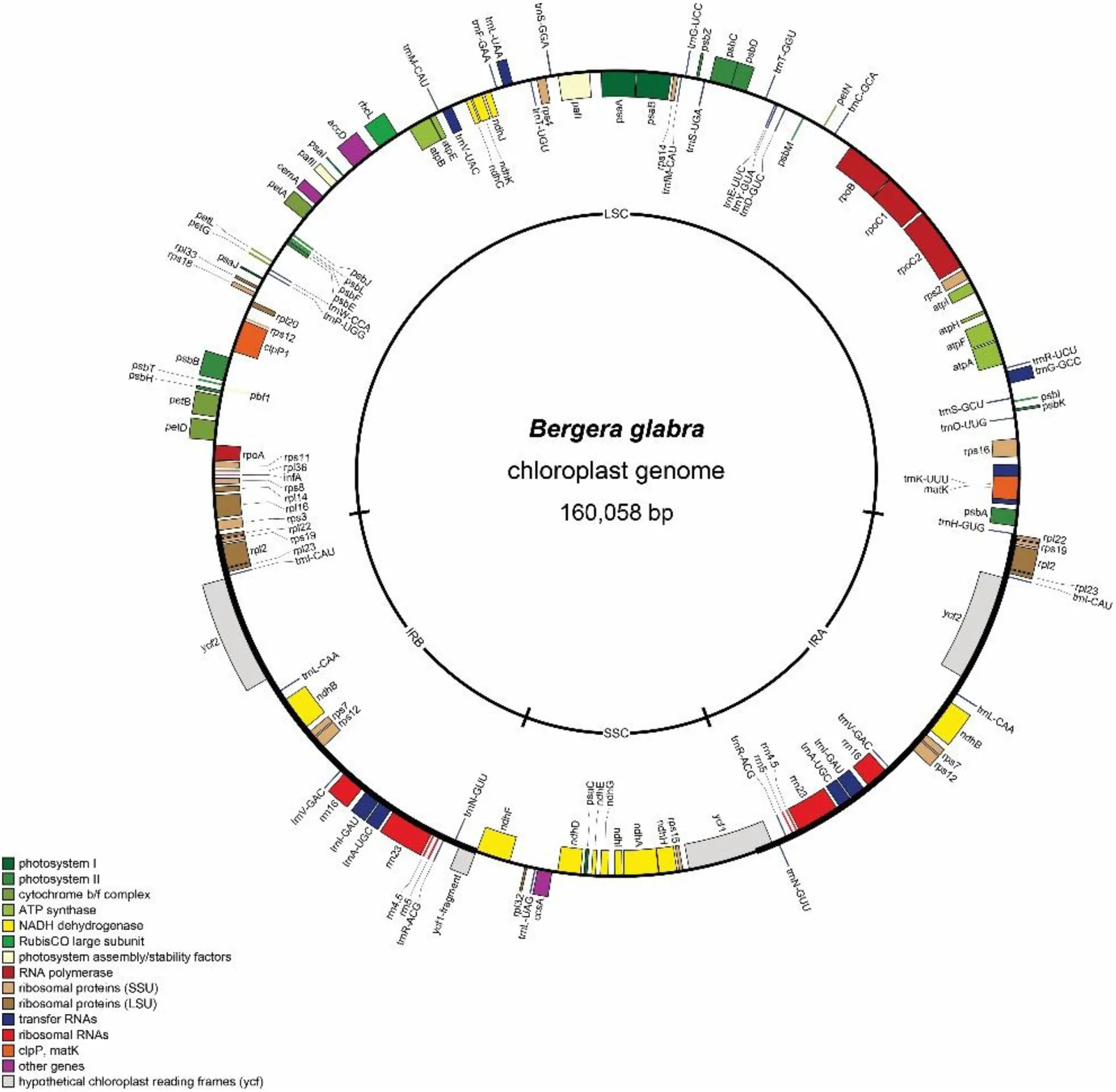
The chloroplast genome map of *Bergera glabra*. Genes located outside the outermost circle are transcribed counterclockwise, while genes inside the circle are transcribed clockwise. Different colors indicate different functional groups of genes. The inner circle represents the locations of four regions in the chloroplast genome. LSC: large single-copy; SSC: small single-copy; IRA and IRB: inverted repeat region A and B, respectively.

The monophyletic status of Rutaceae subfamilies was revealed with high support values (bootstrap value = 100, posterior probability = 1) from both maximum likelihood and Bayesian inference methods (Fig. 4). Similarly, the monophyly of the genus *Bergera* was highly supported. Within the genus *Bergera, B. glabra* was found to be closely related to *B. euchrestifolia*.

**Fig. 4:**
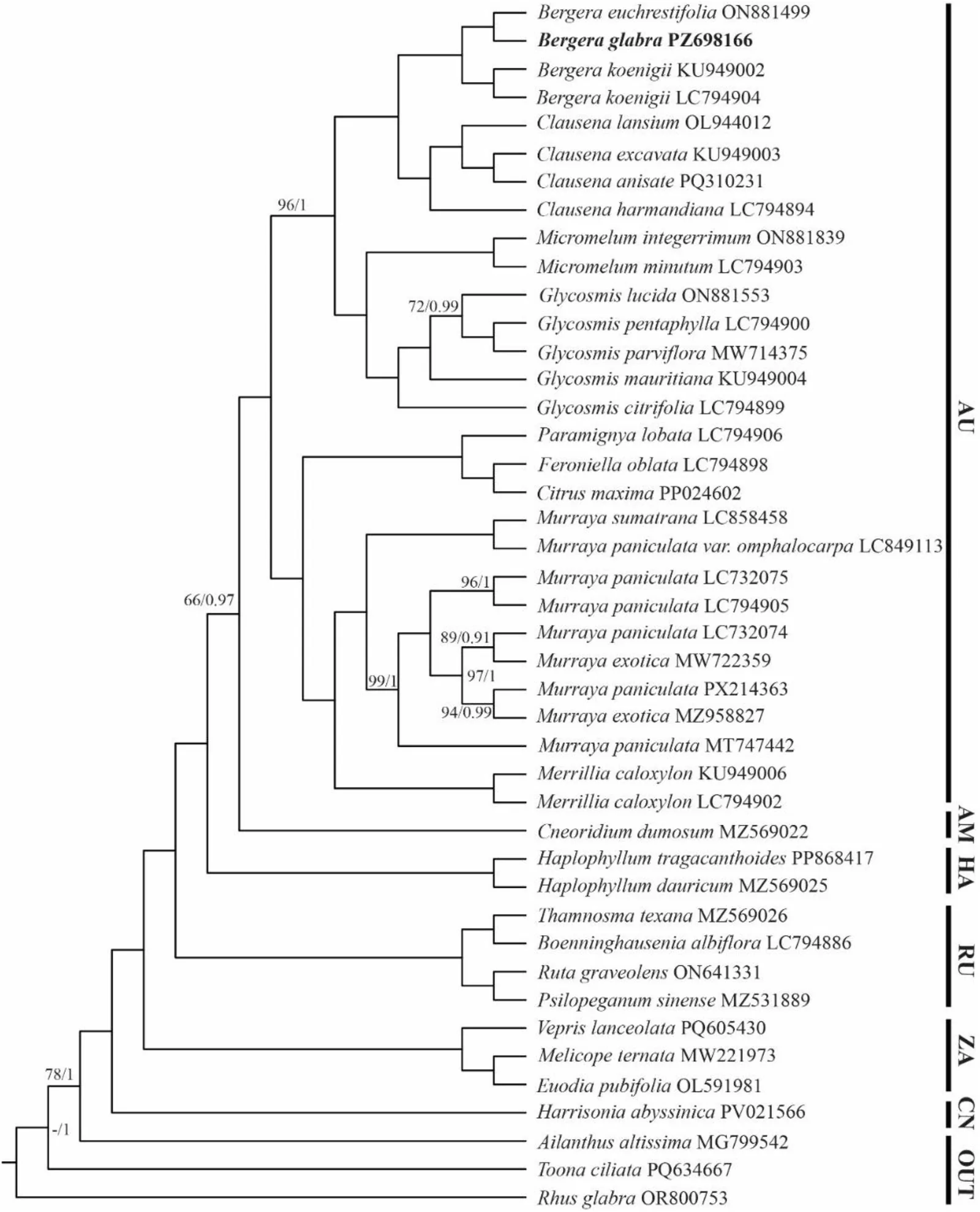
Phylogenetic tree in cladogram format of *Bergera glabra* and related Rutaceae species inferred from the whole chloroplast genome using maximum likelihood and Bayesian inference methods. Bold name indicates target species in the current study. Only bootstrap values below 100 and posterior probabilities below 1 are displayed at the nodes of the phylogenetic tree. Numbers following the species names represent GenBank accession numbers. AU: Aurantioideae, AM: Amyridoideae, HA: Haplophylloideae, RU: Rutoideae, ZA: Zanthoxyloideae, CN: Cneoroideae, OUT: Outgroup.

## 4 Experimental Design, Materials and Methods

### 4.1 Plant sampling, DNA extraction, and next-generation sequencing

The whole plant of *B. glabra* was transplanted from Quang Ninh province to Vinh Long province and was planted at the Medicinal Plant Garden of Tra Vinh University, Vinh Long, Vietnam (9°55′24.7″N 106°20′47.4″E, altitude: 1–2 m) (Fig. 1). The specimen of *B. glabra* was deposited in the School of Agriculture and Aquaculture, Tra Vinh University, under voucher number NHTI-NTTU-20,251,206-003 (contact person: Nguyen Nhat Nam, email: nnnam@tvu.edu.vn). Healthy leaves were collected for extracting total genomic DNA using the DNeasy Plant Pro Kit (Qiagen, USA) following the manufacturer’s protocol. The quality of the DNA sample was checked using a NanoDrop One Microvolume UV–Vis Spectrophotometer (Thermo Fisher Scientific, USA) and 1% agarose gel electrophoresis. The qualified DNA sample, with a minimum concentration of 50 ng/µL and a clear band on the agarose gel, was then sequenced using the NovaSeq sequencing system (Illumina, USA) after library preparation following the manufacturer’s instructions to generate paired-end reads of 150 bp. The sequencing process was conducted at NOVOGENE (Singapore).

### 4.2 Assembly and annotation of chloroplast genome

The reads containing adapters and more than five N bases, as well as those with a Qscore under 20, were discarded from the raw reads (including 439,575,678 reads; ∼ 65.93 Gb; 150 bp in length) using fastp v1.3.6 with default settings [10]. The complete chloroplast genome was then assembled using the filtered reads (including 439,575,678 remaining reads, as no reads had lower quality than the criteria of the filtration step) and NOVOPlasty v4.3.5 with the reference sequence of *Bergera koenigii* (LC794904) and default settings [11]. The coverage depth of the newly assembled chloroplast genome was calculated and visualized using a specific protocol [12]. The gene content of *B. glabra* cpDNA was annotated using GeSeq website with the selection of plastid (land plants), tRNAscan-SE v2.0.7, and chloroplast land plants (CDS+rRNA) of the MPI-MP Reference Set (https://chlorobox.mpimp-golm.mpg.de/geseq.html) [13]. Geneious Prime v2026.0 was used to verify annotations of protein-coding genes for the start and stop codons. OGDRAW v1.3.1 was employed to draw the map of the chloroplast genome with default settings (https://chlorobox.mpimp-golm.mpg.de/OGDraw.html) [14].

### 4.3 Phylogenetic analysis

A total of 43 cpDNAs, including 40 Rutaceae species and three outgroups (*Ailanthus altissima* (Mill.) Swingle of Simaroubaceae, *Toona ciliata* M.Roem. of Meliaceae, and *Rhus glabra* L. of Anacardiaceae), were downloaded from the GenBank database and used to create a data matrix for phylogenetic analysis. The selected 40 species represent six subfamilies of Rutaceae, including Aurantioideae, Amyridoideae, Haplophylloideae, Rutoideae, Zanthoxyloideae, and Cneoroideae, while the three outgroups belong to closely related families of Rutaceae within the order Sapindales. Additionally, within Aurantioideae, representative species of the genus *Bergera*, genus *Murraya*, and related species of Clauseneae and Citreae tribes were selected to confirm the taxonomic status of *B. glabra*. MUSCLE v5 was used to align the complete cpDNAs with default settings [15]. The aligned sequences were then used to reconstruct phylogenetic trees using the maximum likelihood (ML) and Bayesian inference (BI) methods through the IQ-TREE website and MrBayes v3.2.7a, respectively [16,17]. jModelTest2 was used to identify the best substitution model of the aligned sequences, which was identified as TVM+I+G model [18]. The TVM+I+G model was then applied for both ML and BI methods. For ML analysis using IQ-TREE, default settings were used; however, settings of 1000 bootstrap alignments and 1000 replicates were used for ultrafast bootstrap and the Sh-aLRT branch test, respectively. For BI analysis using MrBayes, two chains and 1000,000 generations with sampling frequency of 1000 were employed to reach split frequency lower than 0.01. Additionally, 25% of the samples were applied for burn-in process. The Figtree v1.4.5 was used to illustrate the phylogenetic tree (https://github.com/rambaut/figtree/releases).

## Limitations

This study reports the first complete chloroplast genome of *B. glabra*; however, the dataset was derived from a single individual that was transplanted from its original habitat. Therefore, future studies should include broader population-level sampling and additional chloroplast genomes to elucidate the evolutionary history and population genetic structure of *B. glabra* and to develop informative molecular markers for this species and its relatives within Rutaceae.

## Ethics Statement

The authors have read and follow the ethical requirements for publication in Data in Brief and confirming that the current work does not involve human subjects, animal experiments, or any data collected from social media platforms.

## CRediT Author Statement

**NNN:** Conceptualization, Data curation, Formal analysis, Writing – original draft, Methodology, Writing – review & editing.
